# Complex formation of potassium salt of highly fatty acid with hemagglutinin protein in influenza virus via exothermic interaction

**DOI:** 10.1016/j.bbrep.2022.101302

**Published:** 2022-06-25

**Authors:** Takayoshi Kawahara, Megumi Sakou, Yukie Fumotogawa, Satoshi Kanazawa, Takemasa Sakaguchi, Isamu Akiba

**Affiliations:** a Research and Development Department, Shabondama Soap Co., Ltd., 2-23-1 Minamifutashima, Wakamatsu, Kitakyushu, 808-0195, Japan; b Department of Chemistry and Biochemistry, The University of Kitakyushu, 1-1 Hibikino, Wakamatsu, Kitakyushu, 808-0135, Japan; c Department of Virology, Institute of Biomedical and Health Science, Hiroshima University, 1-2-3 Kasumi, Minami-ku, Hiroshima, 734-8551, Japan

**Keywords:** Natural soap, Influenza virus, Hemagglutinin protein, Small-angle X-ray scattering, Isothermal titration calorimetry

## Abstract

In our previous study, we found highly fatty acid salts, which are a skin-friendly soaps, had a high ability to inactivate the influenza virus. In order to elucidate the mechanism of inactivation of influenza virus, we investigated interactions and complex formation of potassium tetradecanoate (C14K) as a highly fatty acid salt with a virus particle (VP) derived from avian influenza virus by using isothermal titration calorimetry (ITC) and small-angle X-ray scattering (SAXS). ITC showed C14K attractively interacted with hemagglutinin protein (HA) which exists in the envelop of VP. SAXS analyses revealed C14K formed highly ordered complex with HA through the attractive interaction. Since the HA is responsible for cell entry events, inactivation of influenza viruses by highly fatty acid salts are derived owing to HA inhibition of influenza viruses through the complex formation. Time-resolved SAXS measurements elucidated the complex formation was completed within 40 s after mixing aqueous solutions of C14K and VP. This result strongly suggests that hand-washing with a highly fatty acid salts is an effective measure to prevent infection with influenza virus without causing rough hands.

## Highlights

• Pottasium salts of fatty acids show negative change of enthalpy of mixing with influenza viruses.
• Fatty acid salts form complexes with hemagglutinin of influenza viruses through electrostatic interaction.
• The electrostatic complex of fatty acid salts and hemagglutinin forms ordered lamellar structures.
• Inactivation of influenza virus by fatty acid salts is related to hemagglutinin inhibition.

## 1 Introduction

Seasonal spread of influenza viruses poses a serious threat to human health [1,2]. In addition to their health impact, influenza pandemics impose a large economic burden [3]. Epizootic influenza virus, such as avian influenza viruses, can be transmitted from animals to humans, resulting significant morbidity to an infected human [[4], [5], [6], [7]].

Influenza virus infection can be prevented by vaccination and can be treated post-infection with anti-influenza drugs. However, owing to antigenic changes and development of drug resistance, these measures can become ineffective. Therefore, hygiene measures such as handwashing are critical for influenza prevention. Although handwashing with ethanol or surfactants is effective in preventing influenza virus infection and many other pathogens [7], frequent handwashing may cause serious damage to the skin [8,9]. Skin damage increases the risks of secondary infections by staphylococci and Gram-negative bacteria [10,11]. Hence, optimal surfactants for handwashing, which should cause no or minimal skin damage, are intensively requested.

Fatty acid salts, surfactants generated from natural fats, have low cytotoxicity and do not cause skin damage [12]. In addition, in our previous reports, we have found that soaps consisting potassium salts of fatty acids can inactivate influenza viruses more efficiently than other surfactants, such as sodium dodecylsulfate [[13], [14], [15]]. Therefore, fatty acid salts such as potassium oleate should become suitable soaps that inactivate influenza viruses without causing skin damage. The efficient inactivation of influenza viruses may be attributable to interactions between fatty acid salts and virions [13]. However, detail mechanism for inactivation of influenza viruses with fatty acid salts have not been clarified. Thus, we studied here the interactions and molecular assembly in the mixtures of influenza viruses with fatty acid salts to elucidate the mechanisms of influenza virus inactivation by fatty acid salts.

## 2 Materials and methods

### 2.1 Materials

Potassium tetradecanoate (C14K) as a fatty acid salt and potassium oleate (C18=1K) as a control of fatty acid salt were purchased from Tokyo Chemical Industry Co., Ltd. (Tokyo, Japan) and used as obtained. Avian influenza virus A/swan/Shimane/499/83 (H5N3) provided by Dr. K. Otsuki (Totttori University, Japan) and propagated in embryonated chicken eggs was used as an influenza virus. Before the H5N3 virus in which RNA was inactivated by UV irradiation was used as virus particle (VP). The VP was dispersed in phosphate buffer solution (PBS) at 15 μg/mL of protein concentration. An influenza hemagglutinin (HA) vaccine, containing the HA proteins of A/California/7/2009 (H1N1) pdm09, A/Texas/50/2012 (H3N2) and B/Massachusetts/2/2012), was purchased from Biken Co. Ltd. (Osaka, Japan). The vaccine was a split vaccine made from purified virus particles and contained more than 90 μg/mL of the HA proteins from three viruses.

### 2.2 Isothermal titration calorimetry (ITC)

ITC measurements were performed at 25 °C using a VP-ITC MicroCal microcalorimeter (Northampton, MA). Aqueous surfactant solutions (1.75 × 10^−1^ mmol/L) were maintained in the ITC cell at 25 °C with stirring at 300 rpm. Aliquots of VP or HA solutions were injected into the ITC cell using a micro syringe. The volume of each injection was 5 μL. The duration of each injection was 14 s, and there was an interval of 250 s to allow for equilibration correction. The heat of dilution was subtracted even through its contribution to total heat was negligibly small. The enthalpy change (Δ*H*) due to interaction were determined from the titration data on the basis of the amount of C14K or C18=1K.

### 2.3 Small-angle X-ray scattering (SAXS)

SAXS measurements were carried out at BL40B2 and BL03XU stations of SPring-8, Japan. A 2 dimensional photon counting detector (Pillatus 2 M, Dectris, Switzerland) was placed at a distance of 1 m from sample position. The wavelength of the incident X-ray was adjusted to 0.10 nm. Exposure time of static SAXS measurements was kept at 300 s. For time-resolved SAXS measurements, a stopped-flow cell with quartz windows (USP-SFM-CD10, Unisoku, Japan) was used as a sample cell. Aqueous C14K and HA solutions were filled into separate syringes on the stopped-flow apparatus. The two solutions were mixed at a ratio of 1:1 by volume. The temperature in each syrinde and cell was maintained at 25 °C. After the stopped-flow mixing with the dead time of 4 msec, successive X-ray exposures were performed with each exposure time of 100 msec and interval of 200 msec.

The obtained 2-dimensional SAXS images were converted to scattering intensity, *I*(*q*), vs the magnitude of scattering vector, *q*, defined as *q* = (4π/*λ*)sin(*θ*/2), where *λ* is the wavelength of incident X-ray (0.1 nm) and *θ* is the scattering angle.

## 3 Results and discussion

**Fig. 1:**
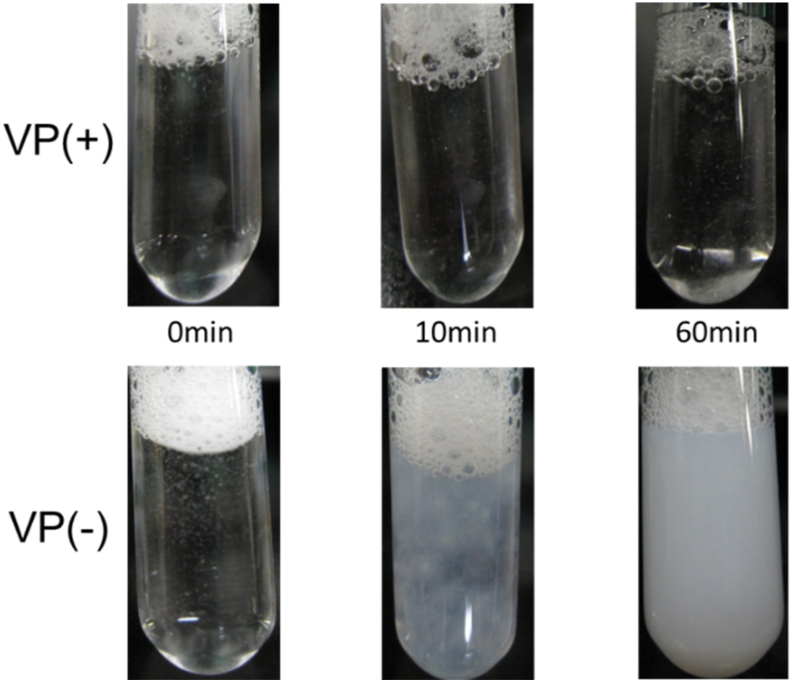
Photographs of changes in states of C14K-VP (VP(+), upper) and C14K-PBS (VP(−), bottom) after mixing of aqueous C14K solution with VP or PBS buffer.

Fig. 1 shows change of the state of aqueous C14K mixed with VP in PBS. When phosphate-buffered saline (PBS), pH 7.4, was added to aqueous C14K solution, precipitates were immediately formed by salting out of C14K (VP(−) in Fig. 1). The amount of C14K precipitates increases with elapsed time. By contrast, no precipitates were formed when PBS containing VPs was added to aqueous C14K (VP(+) in Fig. 1). Thus, the presence of VPs in PBS prevented salting out of C14K, suggesting a strong interaction between C14K and VPs. To study the interactions between C14K with VP, the enthalpy changes of mixing (Δ*H*) the these surfactants with VPs and hemagglutinin protein (HA) were measured by ITC, where HA is a membrane protein of VP responsible for recognition of cell receptors [16].

**Fig. 2:**
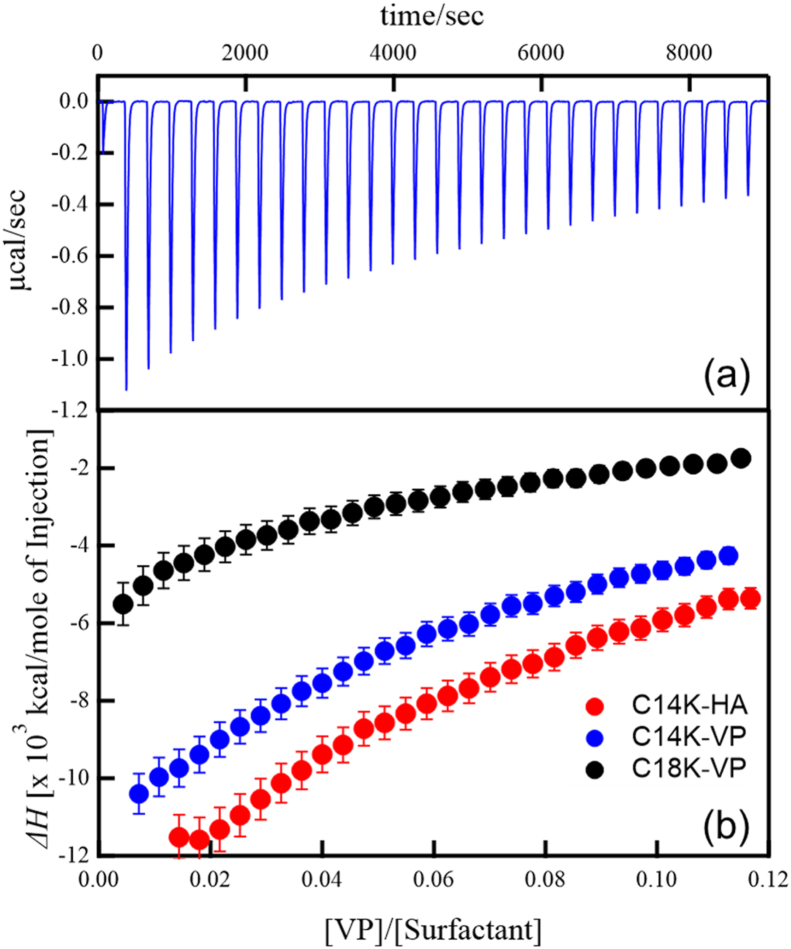
(a) ITC thermograms of C14K-VP mixture and (b) enthalpy change of mixing of C14K-VP (blue), C14-HA (red) and C18=1K-VP (black) mixtures. (For interpretation of the references to colour in this figure legend, the reader is referred to the Web version of this article.)

**Fig. 3:**
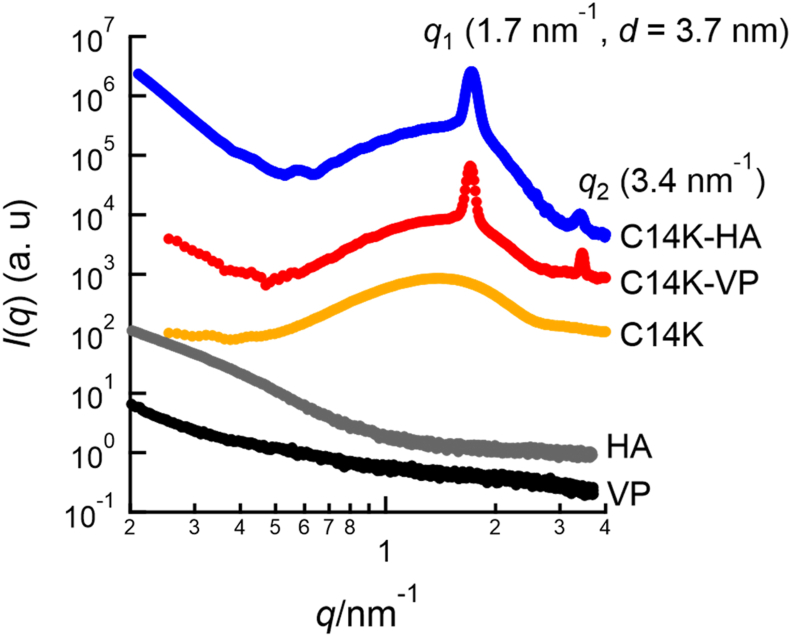
SAXS profiles of C14K-VP mixture, C14K-HA mixture, C14K micelle, HA and VP.

Fig. 2 (a) and (b) show isothermal titration of C14K-VP mixtures and enthalpy change of mixing (Δ*H*) obtained by ITC for C14K-VP, C18=1K-VP and C14K-HA mixtures as a function of volume ratio of VP (or HA) solution to surfactant solution, respectively. The binding isotherms were determined by injecting either VP or HA solution into the C14K or C18=1K solution. Mixtures of C14K or C18=1K and VP showed negative Δ*H*, indicating attractive interactions between potassium salts of fatty acids and VP. In our previous study, we reported that the C18=1K-VP mixtures showed the negative Δ*H* (exothermal change). Thus, a negative Δ*H* appears to be a universal consequence of mixing influenza viruses with potassium salts of fatty acids. On the contrary, our previous paper reported sodium dodecyl sulfate (SDS)-VP and sodium laureth sulfate (LES)-VP mixtures showed positive Δ*H* [13]. The positive Δ*H* indicates fusion of envelop membrane of VP [17,18]. Therefore, the interaction between fatty acid salts and VP can be considered to be different from SDS or LES and VP. The negative Δ*H* indicates the attractive interactions, such as electrostatic interactions or hydrogen bonding, between fatty acid salts and VP [19,20]. The attractive interaction of fatty acid salts with the VP is a factor of efficient inactivation of the influenza virus compared to other surfactants [13]. It is of great interest that which component in VP attractively interacts with C14K or C18=1K. Such attractive interactions do not occur between the fatty acid salts and the envelop membrane consisting of phospholipids. Consequently, the attractive interaction between fatty acid salts and VP should be attributed to the interaction with the spike proteins in the envelope. It has been well-known that there are two spike proteins, HA and neuraminidase (NA), that cause influenza infection. Among them, NA is inhibited by strong binding to a cationic compound having an amino group, which has the opposite charge to the anionic fatty acid salts [[21], [22], [23]]. Hence, it is considered that an attractive interaction does not occur between NA and the fatty acid salts. On the other hand, HA is known to have an attractive interaction with an anionic compound having a carboxy group [24]. Therefore, the exothermic interaction between fatty acid salts and VP is considered to act between HA and fatty acid salts. As shown in Fig. 3, the mixtures of C14K or C18=1K and HA showed intensively negative Δ*H* similar to mixtures of C14K of C18=1K and VPs. Therefore, the attractive interaction between fatty acid salts and VP was attributed to binding of fatty acid salts to HA in the VP envelope. Since the attractive interaction of fatty acid salts with VP is related to effective inactivation of the influenza virus [13], it is considered that the attractive interaction between fatty acid salts with HA plays an important role of effective inactivation of the influenza virus. The amino group at the exposed N-terminus of HA is considered to interact attractively with carboxy group [24]. Thus, inactivation of influenza viruses by potassium salts of fatty acids should be associated with HA inhibition. Binding of C14K to HA should result in the formation of a molecular assembly consisting of C14K and HA. To confirm formation of a molecular assembly in the C14K-VP mixture, we performed SAXS measurements.

**Fig. 4:**
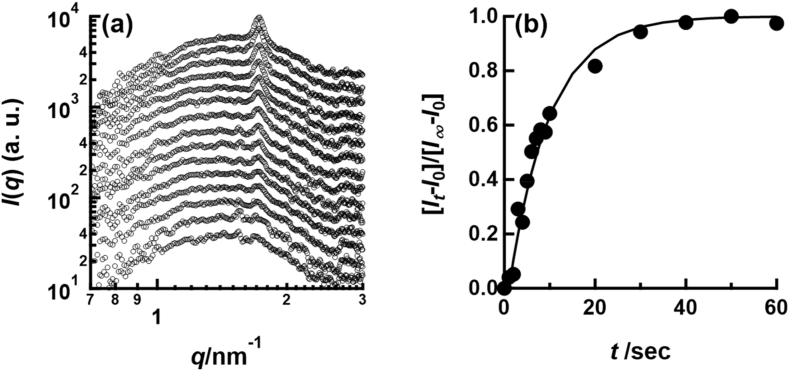
(a) Change in SAXS profile of C14K-VP mixture with time after mixing C14K and VP. (b) Plot of intensity of 1st order peak in SAXS profile as a function of time after mixing of C14K and VP. The solid line is calculated by equation (1) with *τ* = 10.5 s.

Fig. 3 shows how the SAXS profiles (*I*(*q*) vs. *q*) changes upon mixing aqueous C14K and VP. In the SAXS profile from C14K, any diffraction peaks are not observed. The broad peak observed in the SAXS profile of C14K is attributed to the form factor of C14K micelle [25]. By contrast, the SAXS profile of C14K-VP mixture shows diffraction peaks at 1.7 nm^−1^ and 3.4 nm^−1^ in addition to the form factor of C14K micelle. The relative *q* positions of the first and second order peaks are 1 : 2. Hence this diffraction pattern is attributed to the organized lamellar structure with 3.7 nm period. This ordered lamellar structure is an emergent property of C14K-VP mixture. The SAXS profile of the C14K-HA mixture also shows two diffraction peaks at the same *q* positions as those of the C14K-VP mixture. Consequently, the ordered lamellar structure is formed by cooperatively assembly of C14K and HA. The formation of ordered lamellar structure is also confirmed by transmission electron microscopy for C14K and HA (Fig. S1 in supporting information). Based on this result in conjunction with ITC analyses (Fig. 2), we conclude that fatty acid salts form complex with HA through electrostatic interaction. Hence, the fatty acid salts act not only as detergents but also as HA inhibitors via binding and cooperative assembly. The rate of formation of the ordered lamellar structure should correspond to the rate of HA inhibition by fatty acid salt. To confirm the growth rate of the ordered lamellar structure, we performed time-resolved SAXS by using stopped flow cell. Fig. 4 (a) and (b) show how the SAXS profile and the intensity of 1st order peak of ordered lamellar structure change with elapsed time after mixing C14K and VP, respectively. The first order peak of the ordered lamellar structure immediately appears and grows over time after mixing C14K and VP. The intensity of the first order peak steeply increases until 20 s after mixing and then the slope gradually decline. After 40 s from mixing C14K and VP, the intensity of diffraction peak is almost constant. Therefore, the complexation of C14K and VP is completed within 40 s. This behavior is in good agreement with the following single exponential function shown as a solid line in Fig. 4(b), as in the first-order reaction.

(1) $$\frac{\left[I_{t}-I_{0}\right]}{\left[I_{\infty }-I_{0}\right]}=1-\text{exp}\left(-t/\tau \right)$$

Here, It is scattering peak intensity at *q* = 1.7 nm^−1^ at time *t* sec., *I*_∞_ is the average intensity of the peak after 50 s, and *τ* is the time constant. The rate constant calculated from the time constant (*τ* = 10.5 ± 2.0 s) is 9.5 × 10^−2^ sec^−1^, indicating rapid assembly of C14 and HA. Thus, C14K can rapidly inhibit influenza virus HA. To sum up, complex of C14K and HA existing in the envelop of VP is immediately formed through the attractive interaction between carboxy group and HA upon mixing C14K and VP. Then, HA, which is important component for binding to cell surface, is removed from envelop of VP. Inevitably, the ability of infection of VP is significantly reduced. Therefore, highly fatty acid salts are the effective agents for preventing the infection of influenza virus.

## 4 Conclusion

Here, we found that potassium salts of fatty acids strongly binds with HA of influenza virus through exothermic interactions, such as electrostatic interaction. This attractive interaction between the fatty acid salt and HA results in rapid molecular assembly into an ordered lamellar structure and inhibition of HA function. The mechanism of HA inhibition by fatty acid salt is considered to be universal for viruses covered with envelope such as SARS-CoV-2. Because the natural soaps consisting of fatty acid salts have low cytotoxicity and are not damaging to skin, handwashing with these products is an effective measure to prevent infection diseases caused by enveloped viruses without adverse effects.

## Funding

The funder (Shabondama Soap Co., Ltd.) provided support in the form of salaries for TK but did not have any additional role in the study design, data collection and analysis, decision to publish, or preparation of the manuscript. This work was also supported by MEXT Promotion of Distinctive Joint Research Center Program (JPMXP0621467946).

## Author contributions

T. K., T. S., and I. A designed research; T. K., M. S., Y. F., S. K., and I. A. performed research; T. S. contributed influenza virus samples; M. S, Y. F., S. K., and I. A. analyzed data; and T. K. and I. A. wrote the paper.

## Declaration of competing interest

The anti-viral effect of soap is patented in Japan (Patent No. 5593572, An anti-viral and washing agent), Russia, Korea and China. A hand soap having potassium salt of fatty acid is sold by Shabondama Soap Co., Ltd. The funder (Shabondama Soap Co., Ltd.) provided support in the form of salaries for TK. This does not alter our adherence to Biochemistry and Biophysics Reports policies on sharing data and materials.

## Data Availability

No data was used for the research described in the article.
