# Supplementary material for: Complex formation of potassium salt of highly fatty acid with hemagglutinin protein in influenza virus via exothermic interaction
Source: Biochem Biophys Rep. 2022 Jun 25;31:101302. doi: 10.1016/j.bbrep.2022.101302 (PMC9240363; doi:10.1016/j.bbrep.2022.101302)
Supplement: Multimedia component 1 [file mmc1.docx]

Supporting information

Complex Formation of Potassium Salt of Highly Fatty Acid with Hemagglutinin Protein in Influenza Virus via Exothermic Interaction

Takayoshi Kawahara^1^, Megumi Sakou^2^, Yukie Fumotogawa^2^, Satoshi Kanazawa^2^, Takemasa Sakaguchi^3^ and Isamu Akiba^2*^

^1^Research Center for Infection Prevention, Shabondama Soap Co., Ltd., 2-23-1 Minamifutashima, Wakamatsu, Kitakyushu 808-0195

^2^Department of Chemistry and Biochemistry, The University of Kitakyushu, 1-1 Hibikino, Wakamatsu, Kitakyushu 808-0135

^3^Department of Virology, Institute of Biomedical and Health Science, Hiroshima University, 1-2-3 Kasumi, Minami-ku, Hiroshima 734-8551

*Corresponding author

**Transmission electron microscopy (TEM).** TEM observation for dried C14K-VP mixture was carried out by using a JEOL JEM-3010 transmission electron microscope at an accelarating voltage of 100 kV. A droplet of C14K-HA solution was placed on a glass plate. A carbon-coated grid was put on the droplet surface to adsorb the C14K-HA. The excess amount of adsorbed samples was sucked up and dried in reduced pressure.

Fig. S1 shows TEM micrograph for dried C14K-HA mixtures. Several domains with ordred lamellar structure are observed. The periodic length of the lamellar structure is ca. 4 nm corresponding to that obtained by SAXS measurements.


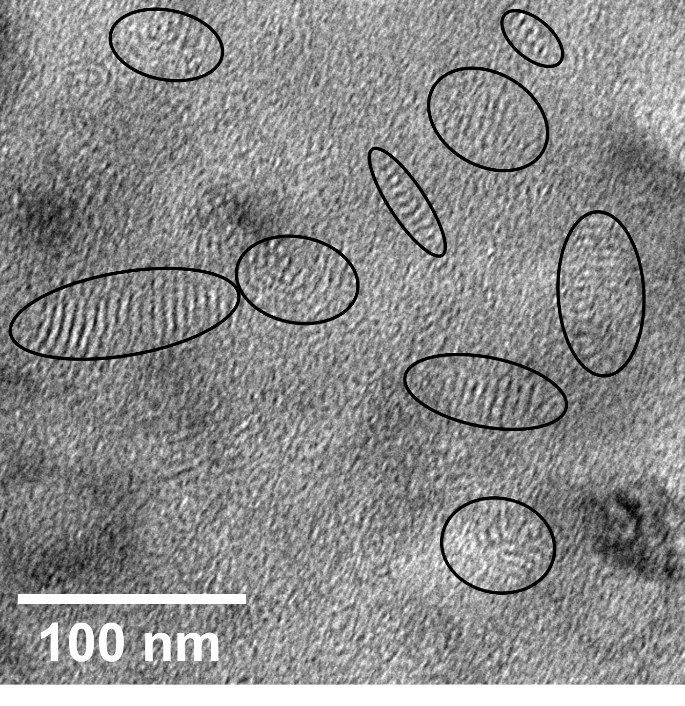


**Fig. S1.** TEM micrograph of C14K-HA mixtures.
